# 

**Published:** 1848

**Authors:** 


### CONTENTS.

### PART I.—ORIGINAL COMMUNICATIONS.

ART. 1 Brainard's Case of Ununited Fracture of the Femur, 187
ART. 2. Rosenkrans' Case of Old Dislocation, 193
ART. 3. Sanders' strictures on the use of Ergot, 195
ART. 4. McGirr on Abdominal Movements, 203
ART. 5. Plummer on Adipose Tumors 206
ART. 6. Stone's Case of Uterine Mole 208

### PART II.—REVIEWS AND NOTICES OF NEW WORKS.

ART. 1. Solly on the Human Brain and Todd and Bowman's Physiology, 212
ART. 2. Burrows on Cerebral Circulation, 228
ART. 3. Taylor on Poisons, 229
ART. 4. Bird's Elements of Natural Philosophy, 231
ART. 5. Paine's Materia Medica, 231
ART. 6. Matteucci's Lectures, 232

### PART III.—SELECTIONS.

ART. 1. Relations existing between the Clerical and Medical prefessions, 233
ART. 2. Naudin on Chronic Amygdalitis, &c., 242
ART. 3. Phillips on Spermatic Discharges, 244
AST. 4. Waller's Case of unavoidable Hemorrhage, 248
ART. 5. Spontaneous Human Combustion, 250
ART. 6. Diagnostic Value of Tears, 251
ART. 8. Fooeign Bodies in the Stomach, 252
ART. 8. Twins born after an interval of two months, 254
ART. 9. Copland on Fever, 253
ART. 10. Garrord on Scurvy, 255
ART. 11. Treatment of Mammary Abscess, 258
ART. 12. Stricture of the Vagina, 259
ART. 13. Coffee as a disinfecting agent 260
ART. 14. Trial of a Physician, 261

### PART IV.—EDITORIAL.

ART. I. Associoation of Superintendents of American Institutions for the Insane, 263
ART. 2. Union Medical Society of Northern Indiana, 269
ART. 3. Iod. Pot. in Hydrocephalus, 271
ART. 4. Case of Severe Pain following Labor, 273
ART. 5. Rush Medical College, 274
ART. 6. Excerpta, 275
ART. 7. Obituary Notice, 276

